# Vertex Stimulation as a Control Site for Transcranial Magnetic Stimulation: A Concurrent TMS/fMRI Study

**DOI:** 10.1016/j.brs.2015.09.008

**Published:** 2016

**Authors:** JeYoung Jung, Andreas Bungert, Richard Bowtell, Stephen R. Jackson

**Affiliations:** a Neuroscience and Aphasia Research Unit (NARU), School of Psychological Sciences, University of Manchester, UK; b WCU Department of Brain and Cognitive Engineering, Korea University, South Korea; c Sir Peter Mansfield Magnetic Resonance Centre, University of Nottingham, UK; d School of Psychology, University of Nottingham, Nottingham, NG7 2RD, UK

**Keywords:** Transcranial magnetic stimulation, Vertex stimulation, Concurrent TMS/fMRI

## Abstract

**Background:**

A common control condition for transcranial magnetic stimulation (TMS) studies is to apply stimulation at the vertex. An assumption of vertex stimulation is that it has relatively little influence over on-going brain processes involved in most experimental tasks, however there has been little attempt to measure neural changes linked to vertex TMS. Here we directly test this assumption by using a concurrent TMS/fMRI paradigm in which we investigate fMRI blood-oxygenation-level-dependent (BOLD) signal changes across the whole brain linked to vertex stimulation.

**Methods:**

Thirty-two healthy participants to part in this study. Twenty-one were stimulated at the vertex, at 120% of resting motor threshold (RMT), with short bursts of 1 Hz TMS, while functional magnetic resonance imaging (fMRI) BOLD images were acquired. As a control condition, we delivered TMS pulses over the left primary motor cortex using identical parameters to 11 other participants.

**Results:**

Vertex stimulation did not evoke increased BOLD activation at the stimulated site. By contrast we observed widespread BOLD deactivations across the brain, including regions within the default mode network (DMN). To examine the effects of vertex stimulation a functional connectivity analysis was conducted.

**Conclusion:**

The results demonstrated that stimulating the vertex with suprathreshold TMS reduced neural activity in brain regions related to the DMN but did not influence the functional connectivity of this network. Our findings provide brain imaging evidence in support of the use of vertex simulation as a control condition in TMS but confirm that vertex TMS induces regional widespread decreases in BOLD activation.

## Highlights

• Combines simultaneous whole-brain fMRI recording with TMS stimulation.
• Investigates the immediate and remote neural correlates of TMS stimulation to the vertex.
• Vertex stimulation leads to widespread decreases in fMRI BOLD, particularly within the brain ‘Default Mode Network’.

## Introduction

TMS has been widely used in neuroscience by producing a magnetic field to generate electrical current in the brain [1]. TMS can establish a causal relationship between brain and behaviours non-invasively by stimulating a specific region and observing TMS induced behavioural changes in measures such as reaction time or accuracy. Researchers have used different control conditions to ensure that the changes in performance are indeed caused by TMS rather than some general factor such as arousal, attention, or the alerting response to the sound of the TMS. One of most common control conditions is to use ‘sham stimulation’. Sham stimulation involves placing the coil away from the scalp [2]. However, a disadvantage of sham stimulation is that it cannot produce the same sensation as experienced when receiving active TMS stimulation. Specifically, when a TMS pulse is discharged, it generates a click sound and a skin sensation is induced by the magnetic field under the coil.

An alternative control condition that is commonly used in the TMS literature is to deliver active stimulation to a brain region that is selected as a control site. This site is invariably assumed to play no active role in the particular brain function under investigation, and the vertex is very often chosen for this reason [3], [4], [5], [6]. Stimulating a control site provides the same sound and the same scalp sensation as TMS stimulation to the targeted region but is assumed to have no functional significance. In particular, the assumption of vertex stimulation is that it does not influence on-going processes involved in task performance. However, there has been little direct evidence to test this assumption. Previous studies using vertex stimulation as their control site have reported task performance changes caused by TMS to the target region but no such changes when the vertex was stimulated [4], [5], [6], [7], [8], [9]. This is not a direct test of the assumption that vertex stimulation does not lead to significant changes in brain activation.

In 1999, Bohning and colleagues demonstrated that it is technically feasible to combine TMS with fMRI recording [10]. By combining TMS with fMRI it is possible to examine how TMS influences the pattern of brain activity in terms of the fMRI BOLD signal. Recent studies using concurrent TMS/fMRI have shown that TMS over primary motor cortex (M1) can evoke BOLD signal changes at the stimulation site and at a number of other remote brain areas [10], [11], [12], [13], [14], [15], [16].

In the current study we tested the assumptions underlying the use of vertex stimulation as a control condition in TMS studies. Specifically, we investigated how vertex stimulation affects neural activity through the whole brain using concurrent TMS/fMRI. We also explored the differing effects of coil orientation over vertex with two orientations, 0° and 180°. To contrast the effects of vertex stimulation, we applied TMS to the left M1 as a control site, and compared the observed patterns of brain activity between these two regions. Finally, based upon our fMRI results, we defined a brain network related to the TMS stimulation and examined the effects of TMS on the functional connectivity within this network.

## Methods

### Participants

Thirty-two healthy adults (6 males; mean age 25 ± 8 years, range 19–30 years, right-handed) participated in the experiment. Twelve participants (3 males; mean age 25 ± 8.3 years, range 20–28 years, one left handed female) performed the experiment when the coil orientation was upright, 0°, and nine subjects (3 males; mean age 25 ± 3.3 years, range 19–30 years) were recruited for the experiment in which the coil orientation was inverted, 180°. Eleven healthy right-handed adults (3 males; mean age 25 ± 3.1 years, range 19–30 years) participated in the experiment in which TMS stimulation was delivered to the left primary motor cortex (M1) as a control site. Handedness was assessed by the Edinburgh handedness inventory [17]. All participants had provided informed written consent in advance of the study. The experiment was approved by a local Research Ethics Committee.

### Experiment design and procedure

**Figure 1:**
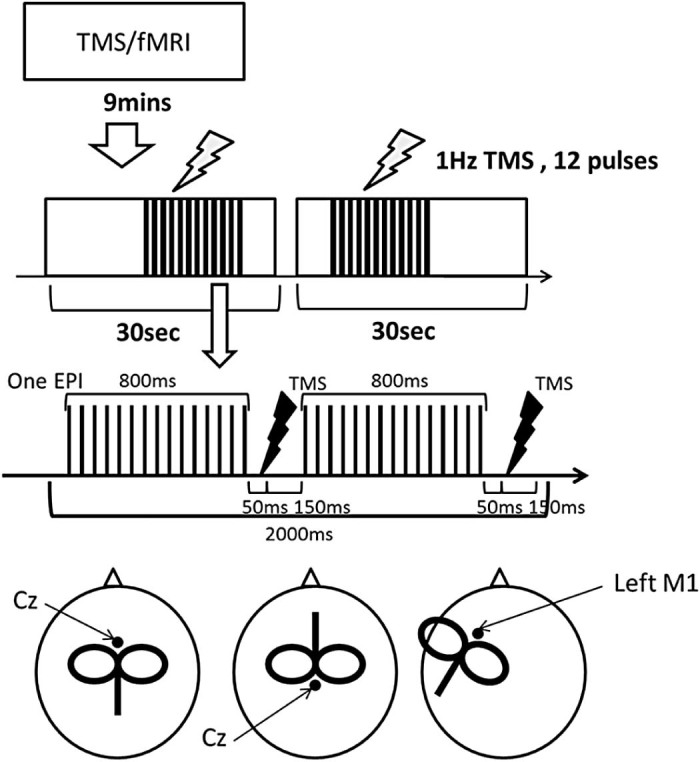
Experimental design and coil locations. Top: in the experiment, there were 18 blocks and each block (30 s) consisted of a TMS phase (11 s) and a No-TMS phase (19 s). Middle: Synchronisation of TMS and fMRI. The figure shows one EPI acquisition consisting of 30 slices. In 1 s, half of the slices are collected leaving a 200 ms gap. The TMS pulse is applied in the gap after the first slice acquisition. Bottom: (left) the vertex TMS coil positioned upright, (middle) the vertex TMS coil positioned inverted, (right) left M1 TMS.

Before the experiment, we measured each participant's RMT outside of the MRI scanner. There were three fMRI experiments, the vertex stimulation (0° and 180°) and the left M1 stimulation. We used a block fMRI design comprising of 18 blocks (9 mins). Each block included a TMS phase (11 s in length) and a No-TMS phase (19 s in length). The occurrence (i.e., temporal onset) of the 11 s sequence of TMS pulses was randomized within each 30 s block. In the TMS phase, 12 pulses of 1 Hz TMS, at 120% of individual RMT, were applied over the vertex or the left M1. All participants were instructed to keep their limbs relaxed through the experiment. The experimental design and procedure is illustrated in Fig. 1.

The following should be noted with respect to the TMS protocol used within our study. We delivered TMS in short 11 s trains during which a single supra-threshold TMS pulse was delivered one each second. This was then followed by a substantial period of no TMS. However, this does not imply that our protocol is an rTMS protocol as commonly conceived. Specifically, it should not, for example, be viewed as an instance of ‘intermittent 1 Hz rTMS’. First the number of TMS pulses delivered in our study is far less than that typically observed in 1 Hz rTMS (i.e. 120 pulses vs. 600 pulses in a 10 minute period). Second, we carried out extensive pilot testing, outside of the MR scanner, prior to our study to determine if our TMS protocol led to any post-stimulation changes in cortical excitability consistent with an rTMS protocol and it did not. For this reason we take the view that our stimulation protocol is best viewed as a set of single TMS pulses and not an rTMS protocol.

### Magnetic resonance imaging (MRI)

MRI was performed at the Sir Peter Mansfield Magnetic Resonance Centre (University of Nottingham) using a Philips 3.0-T scanner equipped with a 6-channel head coil with a detachable front part and a large diameter that provided sufficient space to accommodate the TMS coil (Nova-medical Head Coil outer diameter: 352 mm; inner diameter: 300 mm; and length 330 mm).

Functional images were obtained using single-shot echo planar imaging (EPI) sequence (repetition time (TR)/echo time (TE) = 2000/35 ms, flip angle 90°, 30 slices, matrix = 64 × 64, 3 mm^3^ resolution). Anatomical images were acquired using 3D MP-RAGE sequence (TR/TE = 8.278/2.3 ms, flip angle 8°, matrix = 192 × 192, 1 mm^3^ resolution) covering the whole brain. During scanning all participants wore ear-plugs with head cushioning using tightly-packed foam inserts to prevent head-movement.

### Transcranial magnetic stimulation

A Magstim Rapid^2^ TMS stimulator (Magstim Company, UK) was used to generate TMS pulses with a MR-compatible figure-of-eight coil (70 mm outer wing diameter). In the vertex stimulation condition, the coil was positioned at the vertex [location Cz as measured by the international 10–20 system [18]]. In the left M1 stimulation, the coil was centred over the optimal scalp site for eliciting muscle twitches in the first dorsal interosseous (FDI) muscle of the right hand and was oriented perpendicular to the central sulcus at a 45° angle from the mid-sagittal line approximately (see Fig. 1).

Individual resting motor threshold (RMT) was measured using the following steps. First, a series of single TMS pulse were applied to identify the optimal scalp site for eliciting a muscle twitch in the right FDI muscle. Second, once identified this site was stimulated by changing the intensity of TMS pulses, and threshold was defined as the minimum stimulator output that induced observable muscle movements in five of ten TMS pulses. The mean value of each individual's RMT was 72%, ranging from 58% to 86%.

A plastic coil holder, placed next to the MRI head coil, was used to position the TMS coil. The coil positions and thresholds were re-checked after the participant was positioned within the MRI scanner.

### Synchronisation TMS and fMRI

Previous studies have demonstrated that if, during the acquisition of MR images, a period of at least 100 ms elapses after each TMS pulse, then subsequent MR images are reliably distortion free [10]. In our paradigm, echo-planar images (30 slices) were acquired in 2 s. Half of these slices were collected in an initial period of 800 ms and acquisition of the second set of slices started 200 ms later. Therefore, to avoid distorted images, we applied a TMS pulse 850 ms after the acquisition of the first slice in the EPI. This synchronisation provided a gap 150 ms between the TMS pulse and the subsequent slice acquisition (Fig. 1), which allowed for unperturbed EPI. An in-house Matlab (R2006b) programme was used to synchronise TMS pulses and EPI signal.

### Data analysis

SPM5 software (Wellcome Department of Imaging Neuroscience, UK) was used to analyse the fMRI data. For each individual, the functional images were realigned, co-registered with the anatomical image, spatially normalized to the Montreal Neurological Institute (MNI) space, and spatially smoothed using a Gaussian kernel (8 mm, full-width half-maximal). Individual contrast images were calculated using a general linear model (GLM) in SPM5. A design matrix was defined comprising of the TMS condition and No-TMS condition. T-contrast images were then defined for each participant and the data were analysed at the group level using a random-effects analysis. The contrast images were entered into a one sample *t* test for the experimental conditions (TMS vs. No-TMS and No-TMS vs. TMS) and the contrast images of each experiment (Vertex stimulation vs. left M1 stimulation) were entered into a two samples *t* test for the experimental conditions (TMS vs. No-TMS and No-TMS vs. TMS). A conjunction analysis was conducted to identify brain areas related to the vertex stimulation regardless of the coil orientation. Statistical significance was set to an initial height threshold of p ≤ 0.005 (uncorrected, Z ≥ 3) and the resulting statistic images were assessed for clusters comprising twenty or more simultaneously activated voxels.

### Functional connectivity analysis

Based on the fMRI results, we identified the default mode network (DMN) and motor network (MN) as those resting state networks primarily affected by TMS stimulation. Using the Functional Connectivity (CONN) Toolbox (http://web.mit.edu/swg/software.htm) we performed a functional connectivity analysis. For the DMN, four regions were found in the conjunction analysis that included: medial frontal gyrus (MFG), posterior cingulate cortex (PCC)/Precuneus, and bilateral inferior parietal lobule (IPL). For the MN, regions of interest (ROIs) were defined for M1 bilaterally. Connectivity analysis for fMRI BOLD signals between ROIs provided ROI-to-ROI connectivity estimates for the experimental conditions (TMS and No-TMS). At the individual level, head movements were also entered as a regressor variable. Before averaging individual data, all voxels were filtered using a band pass filter (0.01 < *f* < 0.08) to decrease the effect of low-frequency drift. The CompCor strategy implemented in the toolbox removed several sources of noise from white matter and cerebral fluid voxels. Functional connectivity (Fisher's Z-transformed Pearson correlation coefficient) among ROIs was averaged for each network.

## Results

There were no reports from any participant of any discomfort encountered during the experiment.

### Vertex stimulation

TMS over the vertex produced no significant regions of increased activation throughout the whole brain regardless of the coil orientation. By contrast, we found significant deactivations in contralateral (right) M1, the right precuneus, right SPL, right primary sensory cortex (S1), bilateral superior frontal gyrus (SFG), medial frontal gyrus (MFG), left inferior frontal gyrus (IFG), left S1, and cingulate gyrus when the coil was applied at 180°. When the coil was positioned upright (0°), there were strong deactivations in the middle frontal gyrus (MFG) and cingulate gyrus SG, occipital gyrus, precuneus and caudate nucleus.

**Figure 2:**
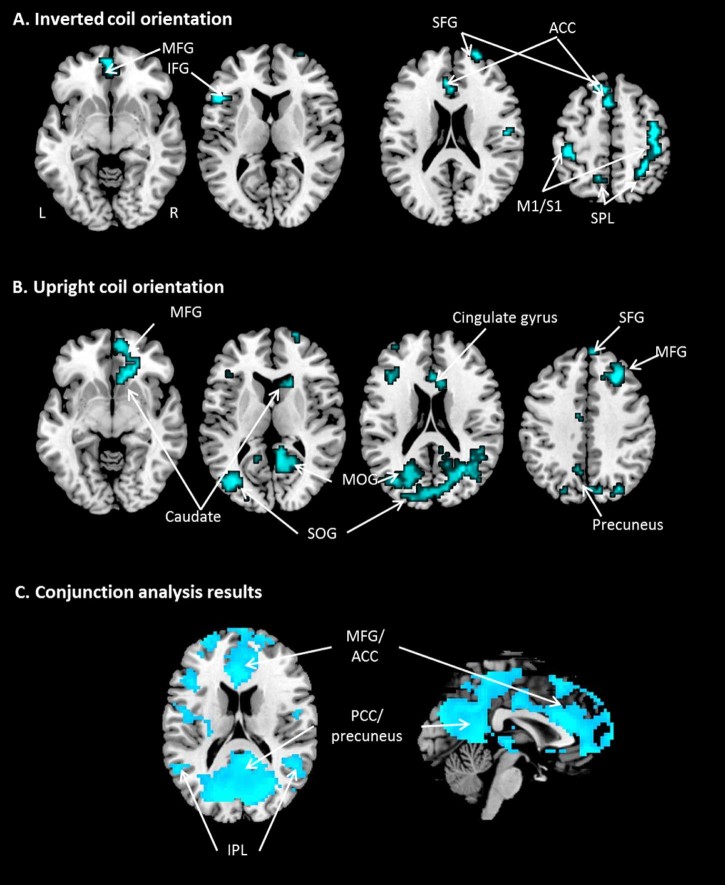
Brain areas that showed significant deactivation following vertex stimulation. (A) Inverted coil orientation; (B) upright coil orientation; (C) conjunction analysis (p < 0.05, FWE). M1: primary motor cortex; S1: primary sensory cortex; SPL: superior parietal lobe; IPL: inferior parietal lobe; SFG: superior frontal gyrus; MFG: medial frontal gyrus; IFG: inferior frontal gyrus; SOG: superior occipital gyrus; MOG: middle occipital gyrus; ACC: anterior cingulate cortex; PCC: posterior cingulate cortex.

To identify the effect of vertex stimulation regardless of the coil orientation we conducted a conjunction analysis. The results demonstrated that vertex stimulation induced significant deactivations in the MFG, anterior and posterior cingulate gyri, inferior parietal lobe (IPL) culmen, cuneus, and precuneus at the level of *p *<* *0.05 (FWE corrected). These regions are associated with the DMN [19], [20]. Again, there were no regions of significantly increased activation induced by vertex TMS. All results are illustrated in Table 1 and Fig. 2.

### Vertex stimulation vs. left M1 stimulation

**Figure 3:**
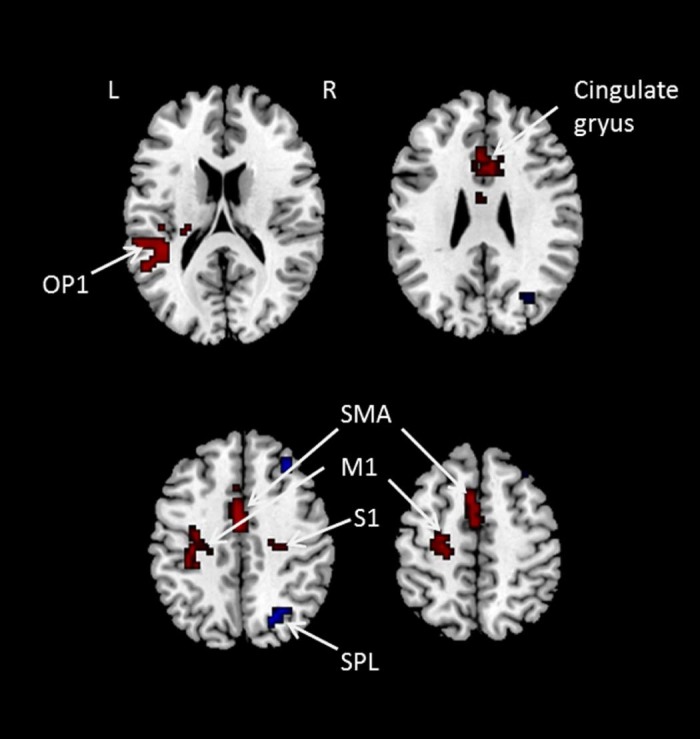
Brain areas that showed activation and deactivation for left M1 stimulation compared to the vertex stimulation. Red colour indicates activation and blue colour indicates deactivation. S1: primary sensory cortex; SMA: supplementary motor area; OP1: Operculum 1; M1: primary motor cortex; SPL: superior parietal lobe. (For interpretation of the references to colour in this figure legend, the reader is referred to the web version of this article.)

TMS delivered to the left M1 compared to the vertex stimulation revealed significant increases in BOLD activation within the left M1, left SII (operculum), left supplementary motor cortex (SMA), cingulate gyrus, and right S1, and significant deactivations in the right precuneus and SPL (Table 2 and Fig. 3).

### Functional connectivity analysis

The fMRI results demonstrated that vertex stimulation evoked significant deactivations in brain regions related to the DMN and that left M1 stimulation induced brain activity changes in regions related to the MN. To examine the effect of TMS on the DMN and MN networks, we employed a functional connectivity analysis. A repeated-measures ANOVA was used with network (DMN vs. MN) and stimulation (TMS vs. No-TMS) as within-subject factors and group (vertex vs. M1) as a between-subject factor. The ANOVA revealed significant main effects of network (F_1, 26_ = 14.14, p = 0.001) and group (F_1, 26_ = 6.88, P = 0.014) and a significant 2-way stimulation × group interaction (F_1, 26_ = 4.99, P = 0.034). Finally, the ANOVA revealed that there was also a statistically significant 3-way stimulation × network × group interaction (F_1, 26_ = 4.20, p = 0.05). All other main and interaction effects were not statistically significant.

**Figure 4:**
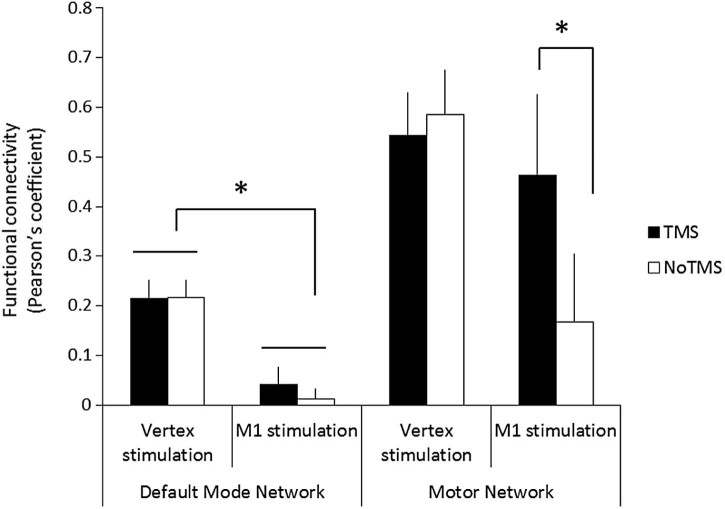
The results of functional connectivity analysis. Black bars represent the TMS condition. White bars represent the No-TMS condition. Error bars represent the standard error. *p < 0.05.

To examine the basis of this 3-way interaction, two separate mixed ANOVAs were conducted for each network with stimulation: (TMS vs. No-TMS) a within-subject factor and group; (vertex vs. M1) a between-subject factor. Results are illustrated in Fig. 4. The ANOVA for the DMN revealed that there was a significant main effect of group (F_1, 26_ = 8.63, P = 0.007) but no significant main effect of condition or a group × condition interaction (p > 0.05).

The ANOVA for the MN revealed that there was a statistically significant group × condition interaction (F_1, 26_ = 5.20, p = 0.031). This effect was further analysed using *post-hoc t* tests that confirmed that, when TMS was applied to the left M1, there was a significant increase in the functional connectivity of the MN (t = 3.36, p < 0.05) which was likely driven by the cortico-cortico disinhibition [21]. This effect was not observed following vertex stimulation.

## Discussion

Vertex stimulation has often been used as a control condition in TMS experiments based upon the assumption that TMS delivered to the vertex has little or no effect on behaviour. Here, we investigated the neural correlates of vertex TMS stimulation using a concurrent TMS/fMRI paradigm that allowed us to investigate changes in the fMRI BOLD signal across the whole brain evoked by short-bursts of TMS. Our findings demonstrated that while there were no significant increases in BOLD activation induced by vertex stimulation using suprathreshold TMS, we nevertheless found that the vertex stimulation evoked significant deactivations across widespread brain areas, including: the MFG, ACC/PCC, IPL, culmen, cuneus, and precuneus. Many of these areas are associated with the DMN [19], [20], [22] which shows a pattern of task-related decrease in precuneus, ACC/PCC, medial prefrontal cortex (mPFC) and IPL. Many researchers have suggested that the DMN is involved in internally focused tasks including: autobiographical memory retrieval, self-related thinking, and consciousness [19]. Our findings indicate that vertex stimulation decreases activation within the DMN which may be attributed to a by-product of TMS. Specifically, TMS produces a skin sensation to the skull together with a loud ‘click’ sound that is delivered with the TMS pulse. It is likely that these additional sensory responses may capture the participant's attention even when at rest, leading to an interruption of the self-related thinking and consciousness attributed to the DMN. It is of interest however that the functional connectivity analysis revealed that vertex stimulation showed relatively higher functional connectivity of the DMN than the M1 stimulation group. As the M1 stimulation with supra-threshold TMS evoked additional muscle movements in the contralateral hand, it may demand more attention or processing resources leading to decreased functional connectivity in the DMN generally. It is also of interest that we found no effect of vertex stimulation on the DMN functional connectivity. It seems contradictive to the increased deactivations of DMN observed when TMS is applied at the vertex. Further studies may be required to investigate this issue. Overall, our finding of no increased fMRI BOLD activity associated with vertex stimulation is broadly consistent with the assumption that vertex stimulation does reliably not influence on-going task performance. However, we acknowledge that further studies may be needed to investigate this issue: particularly studies that investigate the influence of vertex stimulation on the performance of cognitive tasks.

The effects of vertex stimulation compared to M1 stimulation also replicated previous findings using brain imaging. Specifically, previous studies that have stimulated the motor cortex using TMS, combined with brain imaging (fMRI/PET), have shown significant BOLD signal changes to both the targeted (ipsilateral) M1/S1, SMA, ACC and to the contralateral M1/S1 and parietal cortex [11], [14], [23], [24]. Our data demonstrated similar findings, with increased activation observed within the stimulated M1/SI, SMA, ACC and also deactivation within the SPL when the left M1 was stimulated (in comparison to vertex stimulation).

In the MN, we observed the strong effect of M1 stimulation generating a disinhibition between respective M1 regions [21]. However vertex stimulation did not influence MN activation. The results from the functional connectivity analysis support the vertex stimulation assumption by demonstrating that there were no observed changes caused by the vertex stimulation. Although the vertex stimulation evoked significant changes in regional activity, the effect failed to influence the network level. The inter-regional connectivity reflecting fluctuations across the time was not disturbed by the vertex stimulation. Therefore, our findings support the use of vertex stimulation as a control condition.

The pattern of the reduction of BOLD signals was different according to the coil orientation at the lower threshold. Thielscher and colleagues [25] demonstrated that the coil orientation contributed to the change of the magnetic field strength and the range of area that the field covers. The changes in the magnetic field induced by the coil orientation may contribute to the pattern of the decreased activity across the whole brain.

In conclusion, this study showed using concurrent TMS stimulation and fMRI whole-brain recording that vertex TMS stimulation did not induce significant increases in fMRI BOLD activation at any voxels throughout the whole brain. This supports the general assumptions associated with vertex stimulation, specifically that this form of stimulation does not interfere with ongoing task-related activity. However, our results also demonstrate that the vertex stimulation does lead to widespread changes in neural activity in brain regions associated with the DMN, the activity of which is known to be anti-correlated which other brain networks such as the so-called ‘salience’ network. This suggests that vertex stimulation should not be seen as entirely inert, and may not always be the ideal control condition for studies that may involve or interact with the DMN.

## Figures and Tables

**Table 1:** Inverted/upright coil orientation (p < 0.005, unc) and conjunction analysis (p < 0.05, FWE) of the vertex stimulation. BA: Brodmann's area.

	Cluster	BA	Coordinates			Z-score	p Value
x	y	z
Inverted coil orientation							
M1	148	4	39	−12	57	3.68	0
Precuneus		7	30	−42	45	3.57	0
SPL		7	27	−51	63	3.57	0
S1	28	42	51	−12	21	3.73	0
SFG	26	10	24	66	15	3.62	0
IFG	26	45	−48	21	9	3.62	0
SFG	55	6	−3	18	57	3.59	0
SPL	28	7	−12	−57	60	3.55	0
S1	25	1	−42	−30	57	3.36	0
MFG	35	10	0	45	−9	3.15	0.001
Culmen	32		0	−48	3	3.02	0.001
Upright coil orientation							
MFG	102	8	27	27	39	4.04	0
		9	30	24	27	3.46	0
Cingulate cortex	768	31	30	−63	18	3.96	0
SOG		19	−30	−84	24	3.76	0
MOG		19	−36	−75	9	3.51	0
MFG	157	6	−21	0	48	3.85	0
		6	−27	9	54	3.73	0
Cingulate cortex	107	32	15	30	−6	3.76	0
MFG		10	24	66	9	3.49	0
MFG		11	9	24	−9	3.32	0
Caudate	28		9	24	6	3.63	0
SFG	49	9	−15	36	33	3.5	0
Cingulate cortex	40	24	9	18	21	3.33	0
MFG	34	9	6	57	39	3.09	0.001
MFG	28	46	−36	27	21	3.02	0.001
Precuneus	21	7	0	−63	51	2.97	0.001
Conjunction analysis							
Cuneus	45	19	−24	−87	24	6.48	0
			−15	−87	24	5.81	0
Precuneus		31	−24	−78	27	5.31	0
Culmen	59		3	−51	3	6.16	0
Cingulate cortex		29	9	−45	15	5.38	0
MFG	37	10	3	48	−9	5.61	0
			9	54	−9	5.45	0
Cingulate cortex	34	24	0	27	18	5.6	0
		32	0	24	27	5.38	0

**Table 2:** The results of M1 stimulation (p < 0.001, unc).

Region	Cluster	BA	Coordinates			Z-score	p Value
x	y	z
Activation							
Cingulate cortex	228	24	−9	−3	33	4.38	0
		32	9	15	33	3.47	0.001
Cingulate cortex	25	24	18	−12	39	3.43	0
S1		3	27	−21	39	3.27	0.001
SMA		6	−3	0	48	3.22	0
STG	97	22	−51	−45	15	3.4	0
OP1			−30	−30	21	3.36	0
			−42	−39	18	3.2	0.001
M1	108	6	−21	−18	63	3.34	0
		4	−30	−18	48	3.3	0
Deactivation							
SPL	50	7	24	−66	45	3.44	0
Precuneus		19	36	−66	36	3.26	0
